# Cost and resource comparison analysis for THA in Switzerland and Austria

**DOI:** 10.1017/S0266462324000321

**Published:** 2024-10-17

**Authors:** Stefan Blümel, Matthieu Hanauer, Alexander Heimann, Moritz Tannast, Joseph M. Schwab

**Affiliations:** 1 Department of Orthopedic Surgery, HFR Cantonal Hospital, University of Fribourg, Fribourg, Switzerland; 2 WU Vienna University of Economics and Business, Wien, Austria

**Keywords:** total hip arthroplasty, cost comparison analysis, hospitalization, arthroplasty register, healthcare cost, healthcare quality, purchasing power

## Abstract

**Objectives:**

Total hip arthroplasty (THA) is an orthopedic intervention that generates substantial costs to national healthcare systems due to the number of interventions and the cost per intervention. We performed a cost comparison analysis in Austria and Switzerland.

**Methods:**

Data from the national joint arthroplasty register in Switzerland and internal information from the national healthcare services in Austria and Switzerland were compared for patient demographics, interventional characteristics, and costs adjusted for inflation and purchasing power from 2015 to 2021.

**Results:**

The average age for primary THA in Austria was from 67.4 to 67.8 years with 55.9–57.2 percent female patients and from 68.5 to 69.3 years with 52.4–53.8 percent female patients in Switzerland. The annual incidence rate for primary THA rose from 210.28/100k to 216.6/100k in Austria and from 212/100k to 250/100k in Switzerland. After correction for inflation, costs were −1.91 percent lower in Austria in 2021 than in 2015 and −2.57 percent lower in Switzerland. After correction for purchasing power, costs were higher in Austria. The average hospital stay after THA in Austria was reduced by 20 percent (11.7 days/2015 vs. 9.4 days/2021) and 25 percent in Switzerland (8.4 days/2015 vs. 6.4 days/2021). Revision rate was 2.5–3.2 percent in Austria and 2.8–3.2 percent in Switzerland.

**Conclusions:**

The patient population was comparable while patients undergoing primary THA in Austria stay longer in hospital and have relatively higher costs when adjusted for currency, purchasing power, and inflation. The use of standardized registers would be helpful to compare outcomes and costs.

## Introduction

Total joint arthroplasty has been shown to provide substantial functional benefit, be cost-effective, and increase quality-adjusted life years (1). Arthroplasty of the lower extremity, specifically total hip arthroplasty (THA) and total knee arthroplasty, have shown satisfying long-term results and are cost effective (2;3). THA was even titled as the “operation of the century” in 2007 by the Lancet because of its track record of success (4).

The countries of the DACH region (Germany–Austria–Switzerland) are known to have some of the highest number of interventions for lower extremity arthroplasty in the world (5;6). On average, the population in these countries is aging while staying active, like in many European countries. The number of arthroplasties has steadily increased – a trend that is predicted to continue in the coming decades (5;6).

To keep patients mobile, support efficient and effective rehabilitation, and to reduce complications created by long waiting periods prior to intervention, efficiency of the entire surgical experience is paramount. The growing demand for THA may impose a supply problem in the future as healthcare resources become more and more limited. These limitations are, in part, due to high costs and a lack of professional healthcare workers. As THA is creating substantial costs to the healthcare system, optimization of the available resources and cost containment are strongly needed to continue to treat as many patients as possible within the confines of existing healthcare resources.

Cost analysis of discrete, specific interventions, such as THA, could be used to further clarify potential for savings and optimization of procedures. Further, comparison of costs between comparable countries may provide additional insights, though this may be difficult due to the heterogeneity of approaches, indications, and materials used as well as structures and variations between healthcare systems (7).

Austria and Switzerland represent two nations where potential comparisons can be made. Both countries demonstrate very similar population size, geographic location, quality and availability of health care, and demographics. The healthcare delivery system in Austria is universally accessible and is predominantly publicly funded. It is offered to all people residing and working in Austria via existing social security institutions (8). Switzerland also has a universal healthcare system but does not offer free state-provided health services. Instead, private health insurance companies provide compulsory “basic” insurance for all persons residing in Switzerland for 3 months or more (9). Federal assistance for “basic” health insurance is available in cases of economic need. Despite the differences in healthcare coverage paradigms, the similarities make both nations attractive comparators for a healthcare socioeconomic study.

The purpose of this study is to perform a comparative economic analysis of primary THA in Austria and Switzerland, with focus on:

1. patient demographics;
2. per episode cost analysis corrected for national income level;
3. per episode duration of acute inpatient hospitalization;
4. reintervention rate following primary THA.

## Material and methods

### Austria

The “Gesundheit Österreich (GÖK)” (10) provides access to a centralized data registry from Austrian health authorities on the number of primary THAs performed in the general population, the average hospitalization following this procedure, revision rates, as well as basic demographic information such as gender and age distribution. There is no annual report with more detailed information such as type of prosthesis used, approach, cementation, or other surgical points. While basic results such as the total number of interventions performed in Austria per year can be found online (11), detailed information such as patients’ age subgroups according to age or gender can be requested as a paid report from the GÖK. We used this possibility to access the in this article used data for the years 2015–2021 (10).

Since 1997, healthcare costs in Austria are reimbursed to hospitals via the Leistungsorientierte Krankenanstaltenfinanzierung (LKF, *“Performance-oriented Hospital Financing”*) point system. Average costs for an intervention (e.g. THA) can be calculated by the Austrian health authorities using the average LKF point value from the applicable year multiplied by the LKF points charged per surgery. In this way, LKF functions like the diagnosis-related group system used in Switzerland and Germany. Unfortunately, Austria does not maintain a comprehensive, publicly accessible register for arthroplasty data.

### Switzerland

The Swiss National Joint Registry (*Schweizerisches Implantat-*Register or SIRIS) (12) compiles detailed information about joint arthroplasty performed in Switzerland, including, for example, approach and materials used, positioning of the patient during the intervention as well as details of revision interventions and complications. SIRIS makes this information available publicly for free via annual reports and includes detailed data on both primary and revision arthroplasty of both the hip and knee. Starting in 2012, Swiss hospitals are obligated to record specific information on all arthroplasties of the knee and hip in the national register (13). Because of this, 90–97 percent of all primary THAs performed in Switzerland are registered in SIRIS (12), with an increasing capture rate each year. It is important to note that in the annual SIRIS report the costs of the interventions are not included.

Detailed data including rehospitalization rate and average hospitalization duration can be accessed via the SpitalBenchmark network (14) and were made available to us through the controlling department of the cantonal hospital of Fribourg/HFR Fribourg. SpitalBenchmark (14), captures a variety of financial and hospitalization parameters from Swiss hospitals and included data from 89 to 96 percent of all primary THA performed in Switzerland in the study period (Table 1).

**Table 1.** Availability of detailed surgery data and cost information of primary THA interventions in Switzerland from 2015 to 2021

Year	THA interventions in CH	Surgery data available	%	Costs info available	%
2015	17,653	15,717	89	10,618	60
2016	18,699	16,865	90	11,569	62
2017	18,880	17,532	93	12,024	64
2018	19,431	17,431	90	12,046	62
2019	20,099	18,999	95	13,251	66
2020	20,285	19,392	96	13,274	65
2021	21,815	20,732	95	14,462	66

Exact information about costs, however, was only accessible for 60–66 percent of THAs. This is mostly due to the practices of private clinics that are not willing to publicly share financial details (Table 1). Direct comparison of the costs created by primary THA in Austria and Switzerland during the stay in hospital including the surgical intervention is complicated by differences in currencies (€ in Austria, CHF in Switzerland), income levels, and other factors. Therefore, to allow a more exact cost comparison analysis, expenditures were corrected for relative purchasing power in the two countries prior to comparison. The resulting purchasing power represented the average total net income of the population in relation to the place of residence, including net income from self-employed and non-self-employed work, capital income, and state transfer payments such as unemployment benefit, child benefit, and pensions (15).

While SIRIS would have allowed an even more detailed comparison including surgical approach, materials used, and distribution of cemented versus uncemented prostheses, the Austrian arthroplasty data are more limited and do not provide this information. In addition, implant type, manufacturer, surgeon, ASA score, and BMI are not included in the Austrian arthroplasty data. Therefore, a detailed comparative analysis of these factors is not feasible between these two countries.

Annual THA rates in both countries were observed and compared to the population size and growth. Due to the COVID pandemic, planned interventions for arthroplasty were strictly limited in 2020 in most European countries, and the effects are still seen in our global healthcare systems. While we include the years 2020 and 2021 in our analysis, we note that the value of the data might be limited and should be viewed in the broader context of a global pandemic.

## Statistical methods

Descriptive statistics from data delivered by the national health authorities including costs, duration of hospitalization, and revision rate were calculated. Excel® (Microsoft©, Redmond WA, USA) was used for data collection and descriptive analysis. Comparison of demographic and perioperative parameters was performed with the Wilcoxon Rank Sum Test using RStudio (R Foundation for Statistical Computing, Vienna, Austria).

## Ethics statement

For this study, no ethical approval was applied for since data are anonymized register data and are public or semipublic available via contact with the corresponding healthcare authority.

## Results

### Patient demographics

Patients receiving a primary THA in the two countries only slightly but statistically significantly differed regarding age and gender. While the average patient was 1.3 years older in Switzerland (p-value = 0.002), Austrian patients were more often female (56.6 percent vs. 53.1 percent, p-value 0.002) (Table 2). These findings were quite consistent during the review period. In addition, the average patient age increased in both countries during the review period. The biggest increase of average age was seen between 2020 and 2021 in Austria (+ 0.2y) and between 2017 and 2018 in Switzerland (+0.4y) while it slightly reduced in 2020 (−0.1y) in Switzerland during the COVID period. In the same period, the mean age of the general population in Austria increased from 42.3 to 43.2 years (16) and in Switzerland, respectively, from 41.4 to 42.1 years (17).

**Table 2.** Patient demographics for primary THA in Austria and Switzerland in 2015–2021

	♀ Patients in %	♀ Patients in %	Average age at intervention	Average age at intervention
Year	AUT	CH	AUT	CH
2015	56.6	52.6	67.4	68.6
2016	56.4	52.9	67.5	68.5
2017	57.1	53.2	67.6	68.5
2018	56.4	53.4	67.5	68.9
2019	55.9	53.1	67.6	69.1
2020	57.2	52.4	67.6	69.0
2021	56.6	53.8	67.8	69.3

Gender distribution showed to be consistent over time in Austria from 2015 to 2021 with only mild fluctuations observed each year. In Switzerland the proportion of female patients slightly increased during this period with women representing 53.8 percent of the patients in 2021 (+1.2 percent compared to 2015) (Table 2).

### Analysis of primary THA rate: evolution and comparison

From 2015 through 2021, both countries showed an overall increase in primary THA regarding the total number of interventions performed in the population (AUT: +7.2 percent/CH: + 23.6 percent) as well as increase of THA incidence per 100k inhabitants/year (AUT: +3.0 percent/CH: +17.9 percent).

Due to the COVID pandemic, numbers of THA were significantly lower in Austria in 2020 with 2,531 fewer interventions (−12.8 percent) performed in 2020 than in 2019. In 2021 this trend reversed but there were still 445 fewer hip prostheses implanted (−2.2 percent) in 2021 than in 2019.

The number of primary THA increased in Switzerland during both years of the pandemic with 20,285 THA (+186 compared to 2019; +1 percent) performed in 2020. In 2021, the number further increased to 21,815 (+1716 compared to 2019; +8.5 percent) representing the year with the highest number of THAs ever performed in Switzerland.

The incidence rate was quite comparable for primary THA in Austria and Switzerland in 2015 (210 vs. 212/per 100k inhabitants). However, 2021 demonstrated a substantial difference with a minimal increase in Austria and an increase in Switzerland from 2015 to 2021 (217 vs. 250/per 100k inhabitants). During our review period, the maximum THA incidence per 100k in Austria was 227, and in Switzerland was 250. Switzerland also demonstrated year-over-year growth in THA incidence per 100k, with the only period of stagnation being 2020 (Table 3). As the population growth in Switzerland was high during the years observed, the growth of the total number was more impressive than the incidence rate development.

**Table 3.** Development of primary THA in Austria and Switzerland in relation to the general population growth rate from 2015 to 2021

Year	THA interventions total number/year		THA incidence per 100k/year		Population growth in total numbers	
	AUT	CH	AUT	CH	AUT	CH
2015	18,052	17,653	210	212	8,584,926	8,327,126
2016	18,553	18,699	213	222	8,700,471	8,419,550
2017	18,960	18,880	216	223	8,772,865	8,484,130
2018	19,996	19,431	227	227	8,822,267	8,544,527
2019	19,793	20,099	223	234	8,858,775	8,606,033
2020	17,262	20,285	194	234	8,901,064	8,670,300
2021	19,348	21,815	217	250	8,932,664	8,738,791

### Correlation with the general demographic development

During the review period, the resident population of Austria increased moderately from 8,584,926 in 2015 to 8,932,664 in 2021, representing a relative growth of 4.0 percent. Comparison with the growth rate of hip arthroplasty in Austria showed that the total amount of primary THA performed was 3.2 percent higher than the population growth, leading to an increased incidence rate per year of +3.0 percent generated by the strong growth from 2015 until 2019 (Table 3).

In the same period, the Swiss resident population grew from 8,327,126 in 2015 to 8,738,791 in 2021, corresponding to a growth of 4.9 percent in 6 years. The total number of primary THA increased 18.7 percent more than the population, leading to an incidence rate per 100,000 per year that was 17.9 percent higher in 2021 than in 2015 (Table 3).

### Analysis of costs of primary THAs

Evolution of costs of primary THA in Austria from 2015 to 2021 showed an increase from 10,045€ to 11,019€ (+974€; +9.69 percent) for the total costs generated during primary hospitalization. In our review period costs peaked in 2017 and 2018 at 11,841€. Cumulative inflation during the period from 2015 until 2021 was 11.6 percent (18), resulting in an overall cost reduction of approximately 2 percent after correction for inflation.

During the same period, the costs for primary THA in Switzerland, which were accessible for about two thirds of all interventions, decreased from 19,236CHF to 18,779CHF (−457CHF; −2.37 percent). The lowest value was reached in 2019 at 18,636CHF. Since total inflation in Switzerland during the 6 year inclusion period was only 0.2 percent (19), the overall cost reduction, indexed to inflation, remains about the same.

The average Swiss purchasing power was nearly two times higher (1.97) that of Austria in 2015 (15). During the 6 years of observation, the Austrian purchasing power significantly increased while the Swiss purchasing power decreased, therefore the ratio reduced to 1.68 in 2021 (Table 4).

**Table 4.** Cost comparison including adjusted cost comparison for primary THA in Austria and Switzerland from 2015 to 2021

Year	Costs primary THA in Austria in €	Costs primary THA in Switzerland in CHF	Relative purchasing power CH-AUT	Adjusted costs primary THA in Switzerland in €	Difference in € AUT-CH
2015	10,045	19,112	1.97	9,692,16	352,84
2016	10,094	18,851	1.88	10,043,17	50.83
2017	11,841	18,761	1.86	10,059,85	1,781,15
2018	11,841	18,658	1.74	10,737,48	1,103,52
2019	11,816	18,126	1.75	10,370,09	1,445,91
2020	11,241	18,448	1.78	10,359,92	881,08
2021	11,019	17,884	1.68	10,637,60	381,40

After adjusting the costs of primary THA in Switzerland to the relative purchasing power, the apparent absolute cost differences for primary THA showed higher costs in Austria from 2015 until 2021 with the maximum in 2017 (+1781.15€). In 2021, average costs of THA remained slightly higher in Austria (+381.4€) (Table 4).

### Analysis of length of hospitalization

Reviewing the average length of hospitalization after primary THA in Austria showed that patients stayed 2.3 days shorter in 2021 (9.4 days) than in 2015 (11.7 days), representing a length-of-stay reduction of 20 percent. However, when comparing Austria’s length of hospitalization to Switzerland’s over the same period, there is a significantly (p-value 0.0006) longer stay after primary THA in Austria with patients returning home from the hospital after about only 6.4 days (−32 percent) in Switzerland in 2021. From 2015 (8.4 days) until 2021, the Swiss patients left the hospital on average 2 days earlier (−25 percent). There was no information available about the discharge disposition (home, skilled nursing facility, etc.) or services the patients needed.

It is interesting to note that the COVID pandemic had no significant effect, positive or negative, on the overall trend for the average length of stay. In both countries, the trend of a reduced length of stay slowly continued from 2019 until 2021.

### Analysis of revision rate

The revision rate is low in Austria and Switzerland. It was between 2.5 and 3.2 percent in Austria during the observational period and 2.8–3.2 percent in Switzerland without any significant difference (p-value 0.07) between the two countries during the observed period. A slight reduction was achieved from 2015 (3.2 percent in both countries) to 2021 (2.5 percent in Austria, 2.8 percent in Switzerland).

## Discussion

To our knowledge, this article provides the first comparative analysis of direct costs and quality parameters of primary THA of two comparable countries using national health and register data. Cost-efficiency and justified use of resources is not only a current concern but will be a major issue in the coming years as the financial burden of delivering health care, while continuing to deliver better and better outcomes, will continue to grow. Therefore, detailed analyses, including comparison of different healthcare systems, surgical approaches, and technical details of standardized interventions will be demanded to optimize the use of resources.

Register information (Switzerland) and data of the national health authorities (Austria) revealed strong demographic similarities in primary THA patients between the two countries. However, Swiss patients were older by a small amount (but statistically significant), and Austrian patients more often female. Both countries also demonstrated a very slight increase in mean patient age year over year, compared to population age, what can be interpreted as the growing part of active seniors in the population.

These characteristics are comparable to patients in other “Organization for Economic Co-operation and Development” (OECD) countries (20–22). OECD nations include 38 countries representing some of the most high-income and developed economies of the world.

Austria and Switzerland, as well as Germany, are for many years among the OECD countries with the highest rates of primary THAs, a continuing trend that can probably partially be explained as the general European and OECD populations continue to age (22–24).

Although the COVID pandemic put pressure on the healthcare systems worldwide, leading to reductions in planned orthopedic interventions in 2020 and 2021 in most countries, we observed that the incidence of primary THA grew about 17.9 percent in Switzerland from 2015 to 2021. By contrast, Austria exhibited a decline in THAs performed in 2021 compared to 2019, the last pre-COVID year. However, when reviewing only the period from 2015 to 2019 (pre-COVID), the growth rate in THAs in Austria was 9.6 percent, while Switzerland demonstrated a 13.9 percent growth rate during that same period.

Cost evolution and comparison of primary THA in the two countries is a crucially important aspect of our analysis. As mentioned above, a direct cost comparison between both countries is not possible due to the different currencies and income levels. Therefore, cost comparison required correction of the costs through application of the relative purchasing power (15). In addition, a detailed cost analysis would have to include a full breakdown of equipment, surgical approach, prosthesis type, and other factors.

The absolute costs of primary THA were and still are higher in Switzerland. However, after adjusting costs to relative purchasing power, the available data showed higher costs for primary THA in Austria than in Switzerland. Furthermore, costs increased in Austria by approximately 974€ (+9.69 percent) over the review period. When accounting for inflation, this represented approximately a decrease of 2 percent in costs during the period observed. After this correction, cost reduction in Austria was comparable to the cost reduction seen in Switzerland of about 457CHF (−2.37 percent), where inflation was much lower and did not exhibit a major effect.

From 2015 to 2021, primary THA caused 0.41 percent to 0.55 percent of all healthcare spendings in Austria, respectively, 0.44 percent to 0.45 percent in Switzerland. This amounts to 181–236 million € and 337–390 million CHF spent on hip arthroplasty in Austria and Switzerland, respectively, during the years observed. During the COVID period 2020 and 2021, total healthcare spendings and the percentage spent on THA costs dropped in Austria. In Switzerland, healthcare spendings on THA increased parallel to the general cost developments of total healthcare expenditures (Table 5).

**Table 5.** Healthcare spendings for primary THA in Austria and Switzerland from 2015 to 2021

	Total costs THA in million €	Total costs THA in million CHF	Total healthcare costs in million €	Total healthcare costs in million CHF	THA costs = % of healthcare spendings	THA costs % of healthcare spendings
Year	AUT	CH	AUT	CH	AUT	CH
2015	181.3	337.3	38,380,2	74,385.0	0.47	0.45
2016	187.2	352.4	39,613,2	77,455.0	0.47	0.45
2017	224.5	354.2	41,178,1	79,643.0	0.54	0.44
2018	236.7	362.5	42,745,5	80,242.0	0.55	0.45
2019	233.8	364.3	44,308,7	82,472.0	0.52	0.44
2020	194.0	374.2	46,571,7	83,311.0	0.41	0.44
2021	213.1	390.1	49,024,2	86,987.0	0.43	0.44

Cost and quality data of individual Swiss hospitals is publicly available from multiple sources including the Federal Statistical Office (14). The limited access to cost and quality information can pose a substantial challenge in creating positive effective change in those areas. For Austrian hospitals, the average length of stay after THA implantation is publicly available online (25) but the data we were able to collect did not allow for any further detailed analysis or interpretation of outcome.

The Swiss national prosthesis register SIRIS has been well established during recent years and included data on 97.4 percent of all primary hip prostheses performed in 2020 (12). Other national arthroplasty registers, which are well established in countries such from Australia (20), Sweden (21), and Denmark (26) could be used in future studies for more detailed comparison of resource utilization with THA.

Worldwide, fast-track surgery for selected patients has led to an overall trend of shorter hospital stays after primary THA during the last decade (27). Outpatient THA is being performed regularly in the United States and Canada, in contrast to Europe, and has been shown to be cost effective, without statistically different complication rates when compared to “traditional” inpatient THA (28–30). Shorter hospital stays have also been observed in Austria and Switzerland, where outpatient arthroplasty is not routinely performed. We observed a reduction in hospital stay of 20 percent in Austria and 25 percent in Switzerland during 2015–2021. Despite the similar percentage decreases in length of stay, there is a statistically significant baseline 31 percent difference between the two countries, with Austrians staying in hospital after primary THA an average of 9.4 days and Swiss 6.4 days in 2021. However, there was no significant difference between the two countries regarding revision rate as a quality parameter. Optimized management of hospital discharge and postoperative mobilization are necessary to free beds as early as possible to accommodate other patients, limit workload of the healthcare professionals as early as possible and reduce direct hospital costs. Examples from other countries have demonstrated that outpatient THA may be performed in selected patients.

While many factors affect early mobilization, and hence discharge of patients, one of the developing factors that has come under greater scrutiny recently is the choice of surgical approach when performing THA. Minimally invasive approaches, such as the anterior approach, allow full mobilization with minimal if any restriction immediately postoperatively, thereby decreasing the length of hospital stays or prolonged rehabilitation (31). While the SIRIS registry from Switzerland contains information on surgical approach, the available data from the Austrian health system does not. Unfortunately, this limits our ability to analyze how much the surgical approach contributes to length of stay in each country, but our clinical experience indicates that approach is certainly a contributing factor to length of stay. Another important factor that would enrich our analysis is an assessment of where patients are placed after primary hip arthroplasty. While some patients are discharged from the hospital directly to their homes, many elderly patients may be discharged to rehabilitation centers or other “step-down” medical-care facilities to continue their rehabilitation before returning home. Not only does this have an influence on the length of stay in the acute hospital setting, but it may also present an additional healthcare cost that is not adequately represented in the complete cost of care for these patients.

When considering the relatively high volume of orthopedic interventions performed in Austria and Switzerland, especially in comparison to other OECD nations, there are several factors to consider. First, both countries have a higher density of practicing surgeons per population than other nations with Austria boasting 91.8 surgeons per 100k population and Switzerland having 51.2 surgeons per 100k population (32). The average in Europe is 36.2 surgeons per 100k population and 8.7 among all the WHO countries worldwide. In addition, both countries have patient populations with relatively high socioeconomic status, which reduces a financial barrier to performing planned medical interventions.

### Limitations of this study

For this study, we included a mere retrospective evaluation of data. No detailed information about the posthospital care of the patients including inpatient rehabilitation is available from the Austrian or Swiss system but would be valuable to tell if patients really return home earlier in one or the other country. Furthermore, as posthospital information is limited, total included costs are probably not correctly displayed in both countries.

The results from the Austrian healthcare authorities are more limited than the information available from the Swiss SIRIS report, especially surgical details are only poorly accessible. However, information about costs of THA in Switzerland was only accessible for about two thirds of the interventions performed as most of the Swiss private clinics do not share this information.

As we analyzed the period 2015–2021, the COVID period strongly influenced the development in Austria and Switzerland. Ongoing evaluation would be necessary to see if the developments described in our study are continuing or are changing.

Direct comparison of the costs was difficult as the two countries included use a different currency; therefore, we converted the values of the Swiss Franc into € for direct comparison, which may represent a potential source of bias. The purchasing power data from the two countries included which was used for this study was made available from a private company and was not identical to the information offered from the OECD (33). Multiple sources are available for purchasing power data, and both collection and reporting methodologies may result in different numbers and conclusions.

## Conclusion

Our analysis shows that while patient populations are largely similar between the two countries, patients undergoing primary THA in Austria stay longer in hospital and have relatively higher costs when controlled for currency, purchasing power, and inflation. More detailed analysis would be possible if more robust cost and outcome data were available from the Austrian healthcare system and if Austria established a national Joint Replacement Registry.

The Austrian health authorities should aim to further reduce the length of stay in the hospital after THA as this would probably directly reduce costs as well as try to make information more publicly available for research use. The Swiss health authorities should try to include the financial information of the private clinics as only about 66 percent of the financial information of THA, mostly of the public hospitals, is accessible. For both countries, the collection of more detailed information after hospital discharge would be helpful to further assess the total costs produced by the intervention.

As the numbers of THA are higher in Austria and Switzerland than in many countries worldwide, functional health assessment and outcome comparison with OECD members states could be helpful to assess if an overtreatment is happening.

## List of abbreviations

AOANJRR: Australian Orthopaedic Association National Joint Replacement Registry
ASA: American Society of Anesthesiologists physical status classification system
BMI: body mass index
COVID: coronavirus disease
DHR: Danish Hip Arthroplasty Register
DRG: diagnosis-related groups
GÖK: Gesundheit Österreich
LKF: Leistungsorientierte Krankenanstaltenfinanzierung
OECD: Organisation for Economic Co-operation and Development
SIRIS: Schweizerisches Implantat-Register
SAR: Swedish Arthroplasty Register
THA: total hip arthroplasty
TKA: total knee arthroplasty
WHO: World Health Organization
